# Supplementary material for: A large-scale genome-wide cross-trait analysis for the effect of COVID-19 on female-specific cancers
Source: iScience. 2023 Jul 29;26(9):107497. doi: 10.1016/j.isci.2023.107497 (PMC10450412; doi:10.1016/j.isci.2023.107497)
Supplement: Document S1. Figures S1–S18 [file mmc1.pdf]

iScience, Volume *26*

Supplemental information

A large-scale genome-wide cross-trait analysis for the effect of COVID-19 on female-specific cancers

Xunying Zhao, Xueyao Wu, Jinyu Xiao, Li Zhang, Yu Hao, Chenghan Xiao, Ben Zhang, Jiayuan Li, and Xia Jiang

	model1	model2	delta_elpd	se_delta_elpd	z	p
1	null	sharing	0.280	0.24	1.100	0.87
2	null	causal	0.250	1.30	0.190	0.58
3	sharing	causal	-0.027	1.10	-0.024	0.49

model	gamma	eta	q
Sharing	NA	0.14 (-0.82, 0.94)	0.04 (0, 0.26)
Causal	0.05 (-0.02, 0.12)	0.01 (-0.9, 0.87)	0.04 (0, 0.25)

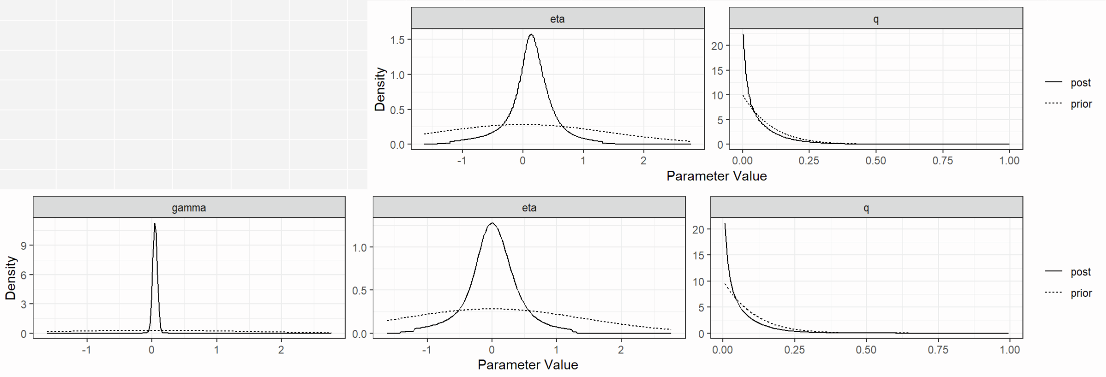

Figure S1. CAUSE-based MR analysis between genetically predicted SRAS-CoV-2 infection and BC risk, related to Figure 2. Model 1 and model 2 refer to the models being compared (null, sharing, or causal). Model fit is measured by Δ Expected Log Pointwise Posterior Density (ELPD); Negative values of z indicate that model 2 is a better fit. Delta_elpd: Estimated difference in ELPD. se_delta_elpd: Estimated standard error of delta_elpd, and z: delta_elpd/se_delta_elpd.

	model1	model2	delta_elpd	se_delta_elpd	z	p
1	null	sharing	-1.8	1.7	-1.10	0.13
2	null	causal	-3.0	2.9	-1.00	0.15
3	sharing	causal	-1.2	1.7	-0.66	0.25

model	gamma	eta	q
Sharing	NA	0.65 (-0.14, 1.28)	0.13 (0.01, 0.34)
Causal	0.14 (0.01, 0.28)	0.28 (-1.93, 1.77)	0.05 (0, 0.26)

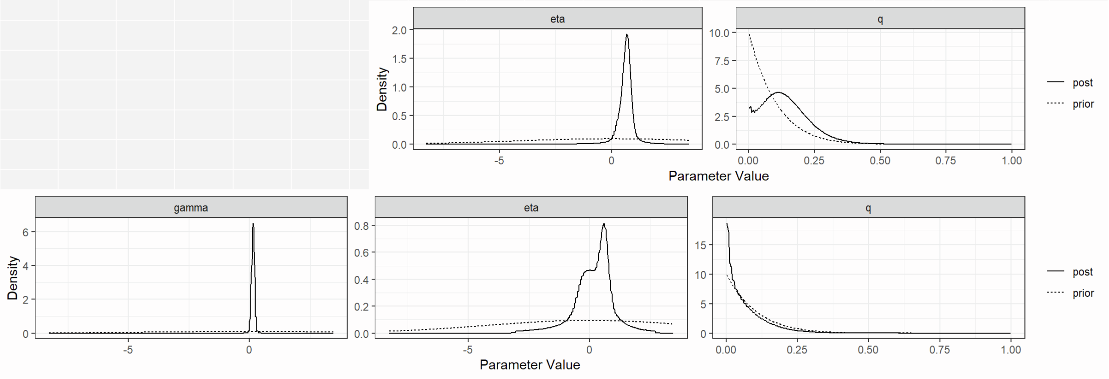

Figure S2. CAUSE-based MR analysis between genetically predicted SRAS-CoV-2 infection and EOC risk, related to Figure 2. Model 1 and model 2 refer to the models being compared (null, sharing, or causal). Model fit is measured by Δ Expected Log Pointwise Posterior Density (ELPD); Negative values of z indicate that model 2 is a better fit. Delta_elpd: Estimated difference in ELPD. se_delta_elpd: Estimated standard error of delta_elpd, and z: delta_elpd/se_delta_elpd.

	model1	model2	delta_elpd	se_delta_elpd	z	p
1	null	sharing	-0.77	1.0	-0.74	0.23
2	null	causal	-1.50	2.3	-0.65	0.26
3	sharing	causal	-0.72	1.4	-0.53	0.30

model	gamma	eta	q
Sharing	NA	0.54 (-1.2, 1.34)	0.1 (0, 0.32)
Causal	0.13 (-0.02, 0.28)	-0.03 (-2.2, 1.4)	0.04 (0, 0.27)

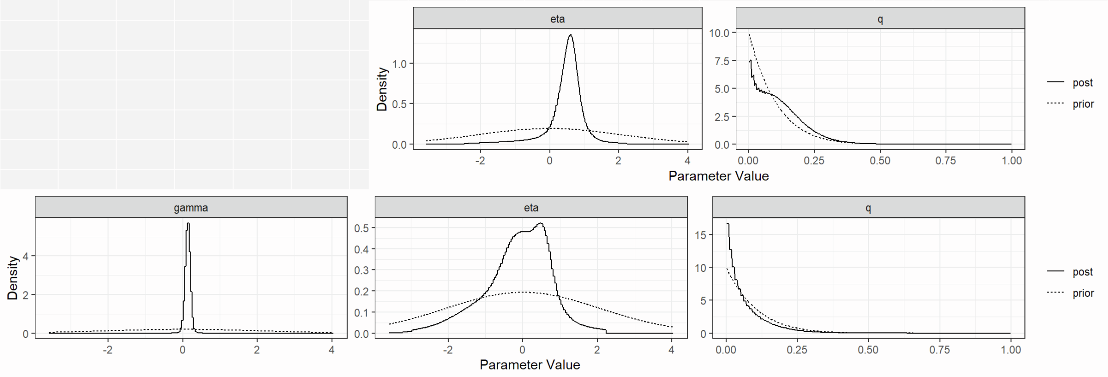

Figure S3. CAUSE-based MR analysis between genetically predicted SRAS-CoV-2 infection and EC risk, related to Figure 2. Model 1 and model 2 refer to the models being compared (null, sharing, or causal). Model fit is measured by Δ Expected Log Pointwise Posterior Density (ELPD); Negative values of z indicate that model 2 is a better fit. Delta_elpd: Estimated difference in ELPD. se_delta_elpd: Estimated standard error of delta_elpd, and z: delta_elpd/se_delta_elpd.

	model1	model2	delta_elpd	se_delta_elpd	z	p
1	null	sharing	0.098	0.43	0.23	0.59
2	null	causal	-0.740	1.80	-0.42	0.34
3	sharing	causal	-0.830	1.40	-0.61	0.27

model	gamma	eta	q
Sharing	NA	0.09 (-0.24, 0.44)	0.05 (0, 0.29)
Causal	0.02 (0, 0.05)	0.02 (-0.34, 0.4)	0.04 (0, 0.25)

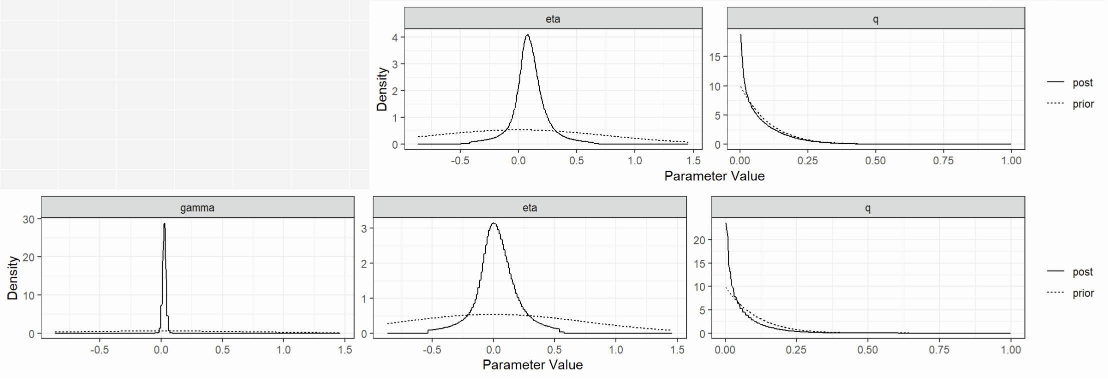

Figure S4. CAUSE-based MR analysis between genetically predicted COVID-19 hospitalization and BC risk, related to Figure 2. Model 1 and model 2 refer to the models being compared (null, sharing, or causal). Model fit is measured by Δ Expected Log Pointwise Posterior Density (ELPD); Negative values of z indicate that model 2 is a better fit. Delta_elpd: Estimated difference in ELPD. se_delta_elpd: Estimated standard error of delta_elpd, and z: delta_elpd/se_delta_elpd.

	model1	model2	delta_elpd	se_delta_elpd	z	p
1	null	sharing	0.47	0.098	4.8	1
2	null	causal	1.50	0.250	5.8	1
3	sharing	causal	0.99	0.210	4.7	1

model	gamma	eta	q
Sharing	NA	0.03 (-0.73, 0.71)	0.03 (0, 0.22)
Causal	0 (-0.05, 0.04)	0.04 (-0.67, 0.66)	0.04 (0, 0.26)

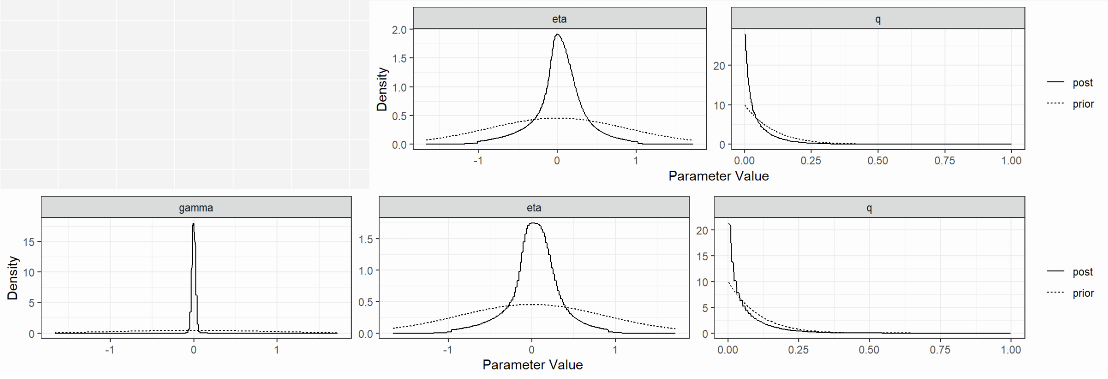

Figure S5. CAUSE-based MR analysis between genetically predicted COVID-19 hospitalization and EOC risk, related to Figure 2. Model 1 and model 2 refer to the models being compared (null, sharing, or causal). Model fit is measured by Δ Expected Log Pointwise Posterior Density (ELPD); Negative values of z indicate that model 2 is a better fit. Delta_elpd: Estimated difference in ELPD. se_delta_elpd: Estimated standard error of delta_elpd, and z: delta_elpd/se_delta_elpd.

	model1	model2	delta_elpd	se_delta_elpd	z	p
1	null	sharing	-3.20	2.8	-1.10	0.13
2	null	causal	-3.40	3.2	-1.10	0.14
3	sharing	causal	-0.22	1.6	-0.14	0.44

model	gamma	eta	q
Sharing	NA	0.5 (0.14, 0.83)	0.08 (0.02, 0.23)
Causal	0.04 (-0.01, 0.09)	0.44 (-0.77, 0.91)	0.04 (0, 0.19)

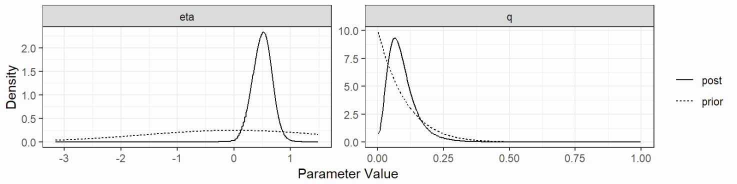

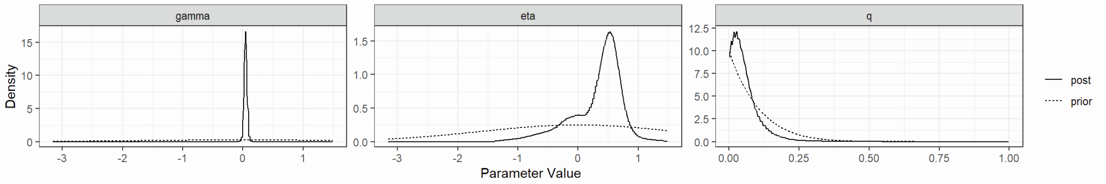

Figure S6. CAUSE-based MR analysis between genetically predicted COVID-19 hospitalization and EC risk, related to Figure 2. Model 1 and model 2 refer to the models being compared (null, sharing, or causal). Model fit is measured by Δ Expected Log Pointwise Posterior Density (ELPD); Negative values of z indicate that model 2 is a better fit. Delta_elpd: Estimated difference in ELPD. se_delta_elpd: Estimated standard error of delta_elpd, and z: delta_elpd/se_delta_elpd.

	model1	model2	delta_elpd	se_delta_elpd	z	p
1	null	sharing	0.49	0.034	14.0	1
2	null	causal	1.10	0.180	6.3	1
3	sharing	causal	0.64	0.170	3.7	1

model	gamma	eta	q
Sharing	NA	0 (-0.34, 0.35)	0.03 (0, 0.22)
Causal	0 (-0.02, 0.02)	0.01 (-0.27, 0.3)	0.03 (0, 0.24)

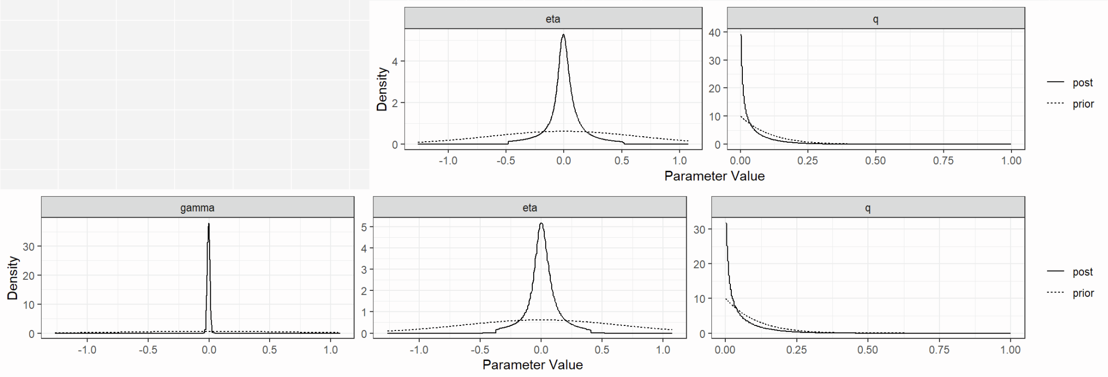

Figure S7. CAUSE-based MR analysis between genetically predicted COVID-19 critical illness and BC risk, related to Figure 2. Model 1 and model 2 refer to the models being compared (null, sharing, or causal). Model fit is measured by Δ Expected Log Pointwise Posterior Density (ELPD); Negative values of z indicate that model 2 is a better fit. Delta_elpd: Estimated difference in ELPD. se_delta_elpd: Estimated standard error of delta_elpd, and z: delta_elpd/se_delta_elpd.

	model1	model2	delta_elpd	se_delta_elpd	z	p
1	null	sharing	0.48	0.11	4.4	1
2	null	causal	1.50	0.25	6.1	1
3	sharing	causal	1.00	0.22	4.6	1

model	gamma	eta	q
Sharing	NA	0.02 (-0.54, 0.52)	0.03 (0, 0.22)
Causal	0 (-0.03, 0.03)	0.03 (-0.51, 0.5)	0.03 (0, 0.26)

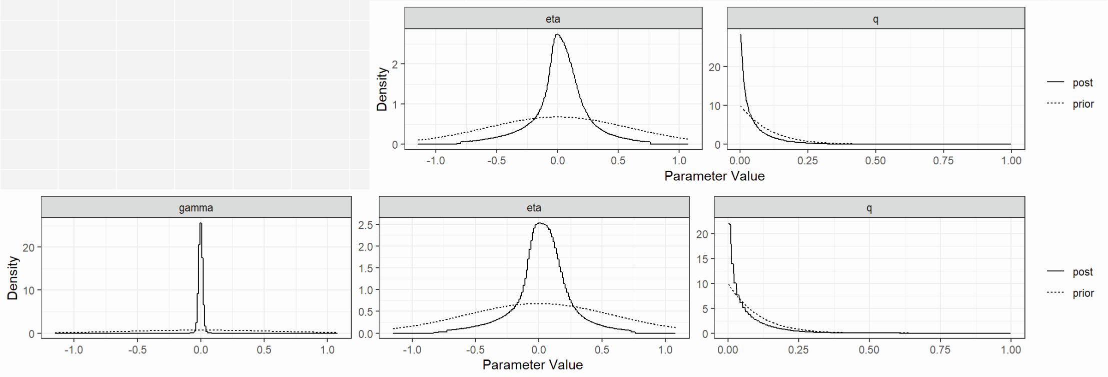

Figure S8. CAUSE-based MR analysis between genetically predicted COVID-19 critical illness and EOC risk, related to Figure 2. Model 1 and model 2 refer to the models being compared (null, sharing, or causal). Model fit is measured by Δ Expected Log Pointwise Posterior Density (ELPD); Negative values of z indicate that model 2 is a better fit. Delta_elpd: Estimated difference in ELPD. se_delta_elpd: Estimated standard error of delta_elpd, and z: delta_elpd/se_delta_elpd.

	model1	model2	delta_elpd	se_delta_elpd	z	p
1	null	sharing	-4.1	2.8	-1.5	0.07
2	null	causal	-6.3	3.9	-1.6	0.05
3	sharing	causal	-2.2	1.9	-1.1	0.13

model	gamma	eta	q
Sharing	NA	0.24 (0.08, 0.48)	0.13 (0.03, 0.36)
Causal	0.05 (0.01, 0.08)	0.1 (-0.59, 0.56)	0.04 (0, 0.22)

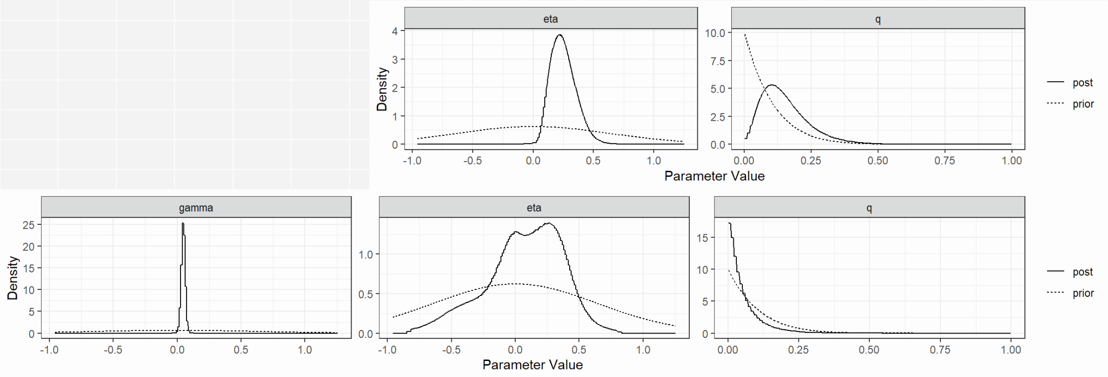

Figure S9. CAUSE-based MR analysis between genetically predicted COVID-19 critical illness and EC risk, related to Figure 2. Model 1 and model 2 refer to the models being compared (null, sharing, or causal). Model fit is measured by Δ Expected Log Pointwise Posterior Density (ELPD); Negative values of z indicate that model 2 is a better fit. Delta_elpd: Estimated difference in ELPD. se_delta_elpd: Estimated standard error of delta_elpd, and z: delta_elpd/se_delta_elpd.

	model1	model2	delta_elpd	se_delta_elpd	z	p
1	null	sharing	0.35	0.25	1.40	0.92
2	null	causal	0.77	1.20	0.66	0.74
3	sharing	causal	0.42	0.95	0.45	0.67

model	gamma	eta	q
Sharing	NA	0.02 (-0.18, 0.2)	0.03 (0, 0.23)
Causal	0.01 (-0.01, 0.02)	0 (-0.11, 0.12)	0.04 (0, 0.26)

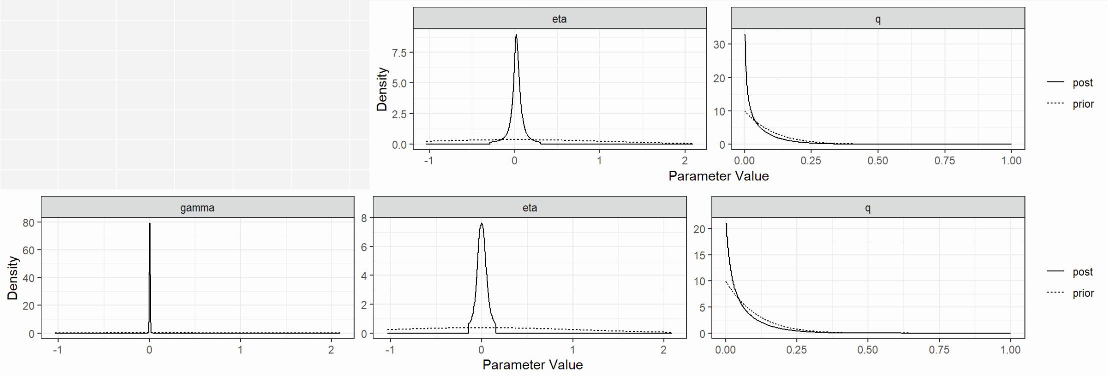

Figure S10. CAUSE-based MR analysis between genetically predicted BC and SRAS-CoV-2 infection, related to Figure 2. Model 1 and model 2 refer to the models being compared (null, sharing, or causal). Model fit is measured by Δ Expected Log Pointwise Posterior Density (ELPD); Negative values of z indicate that model 2 is a better fit. Delta_elpd: Estimated difference in ELPD. se_delta_elpd: Estimated standard error of delta_elpd, and z: delta_elpd/se_delta_elpd.

	model1	model2	delta_elpd	se_delta_elpd	z	p
1	null	sharing	0.56	0.15	3.7	1
2	null	causal	1.50	0.18	8.6	1
3	sharing	causal	0.97	0.19	5.1	1

model	gamma	eta	q
Sharing	NA	0.01 (-0.41, 0.47)	0.03 (0, 0.21)
Causal	0 (-0.02, 0.02)	0.03 (-0.38, 0.42)	0.03 (0, 0.24)

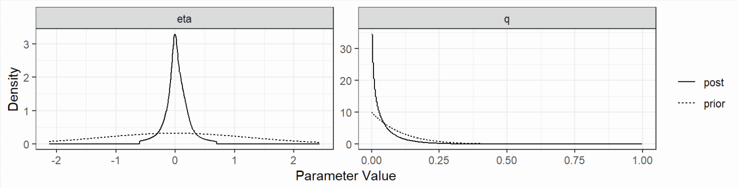

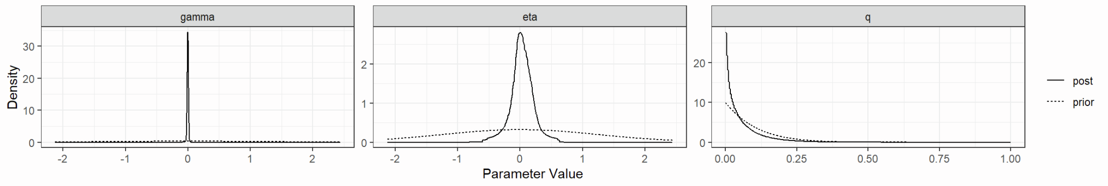

Figure S11. CAUSE-based MR analysis between genetically predicted BC and COVID-19 hospitalization, related to Figure 2. Model 1 and model 2 refer to the models being compared (null, sharing, or causal). Model fit is measured by Δ Expected Log Pointwise Posterior Density (ELPD); Negative values of z indicate that model 2 is a better fit. Delta_elpd: Estimated difference in ELPD. se_delta_elpd: Estimated standard error of delta_elpd, and z: delta_elpd/se_delta_elpd.

	model1	model2	delta_elpd	se_delta_elpd	z	p
1	null	sharing	0.21	0.43	0.50	0.69
2	null	causal	0.38	1.50	0.25	0.60
3	sharing	causal	0.17	1.10	0.15	0.56

model	gamma	eta	q
Sharing	NA	-0.1 (-0.71, 0.59)	0.04 (0, 0.26)
Causal	-0.02 (-0.06, 0.01)	-0.01 (-0.68, 0.76)	0.03 (0, 0.26)

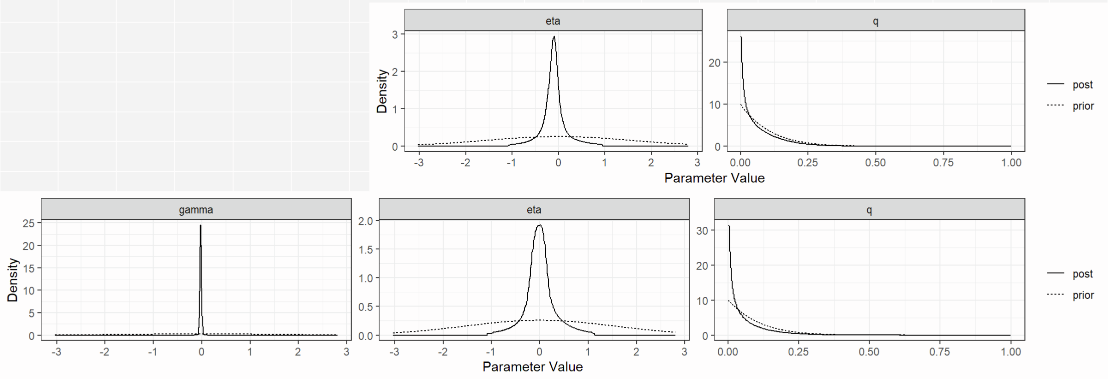

Figure S12. CAUSE-based MR analysis between genetically predicted BC and COVID-19 critical illness, related to Figure 2. Model 1 and model 2 refer to the models being compared (null, sharing, or causal). Model fit is measured by Δ Expected Log Pointwise Posterior Density (ELPD); Negative values of z indicate that model 2 is a better fit. Delta_elpd: Estimated difference in ELPD. se_delta_elpd: Estimated standard error of delta_elpd, and z: delta_elpd/se_delta_elpd.

	model1	model2	delta_elpd	se_delta_elpd	z	p
1	null	sharing	0.50	0.076	6.6	1
2	null	causal	1.20	0.130	9.4	1
3	sharing	causal	0.68	0.120	5.7	1

model	gamma	eta	q
Sharing	NA	0.01 (-0.27, 0.66)	0.02 (0, 0.22)
Causal	0 (-0.03, 0.02)	0.01 (-0.23, 0.63)	0.03 (0, 0.24)

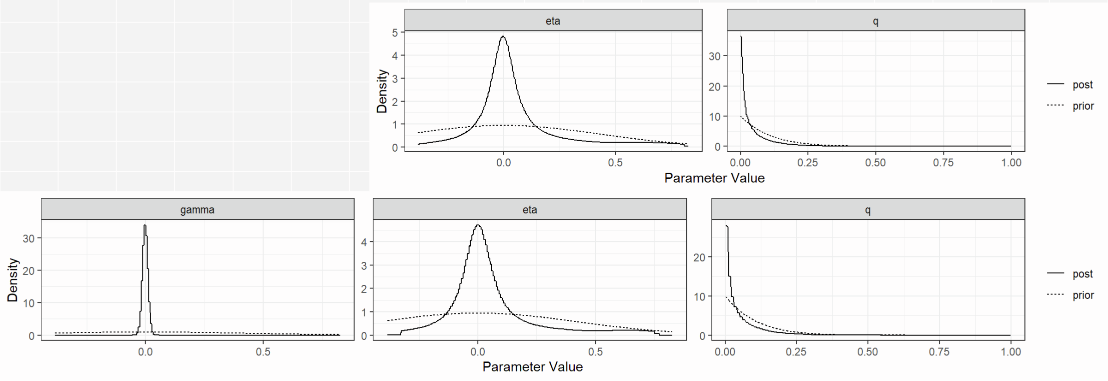

Figure S13. CAUSE-based MR analysis between genetically predicted EOC and SRAS-CoV-2 infection, related to Figure 2. Model 1 and model 2 refer to the models being compared (null, sharing, or causal). Model fit is measured by Δ Expected Log Pointwise Posterior Density (ELPD); Negative values of z indicate that model 2 is a better fit. Delta_elpd: Estimated difference in ELPD. se_delta_elpd: Estimated standard error of delta_elpd, and z: delta_elpd/se_delta_elpd.

	model1	model2	delta_elpd	se_delta_elpd	z	p
1	null	sharing	0.37	0.053	7.00	1.00
2	null	causal	0.71	0.650	1.10	0.86
3	sharing	causal	0.34	0.610	0.55	0.71

model	gamma	eta	q
Sharing	NA	-0.04 (-0.51, 0.48)	0.04 (0, 0.25)
Causal	-0.03 (-0.09, 0.03)	0.01 (-0.45, 0.5)	0.04 (0, 0.26)

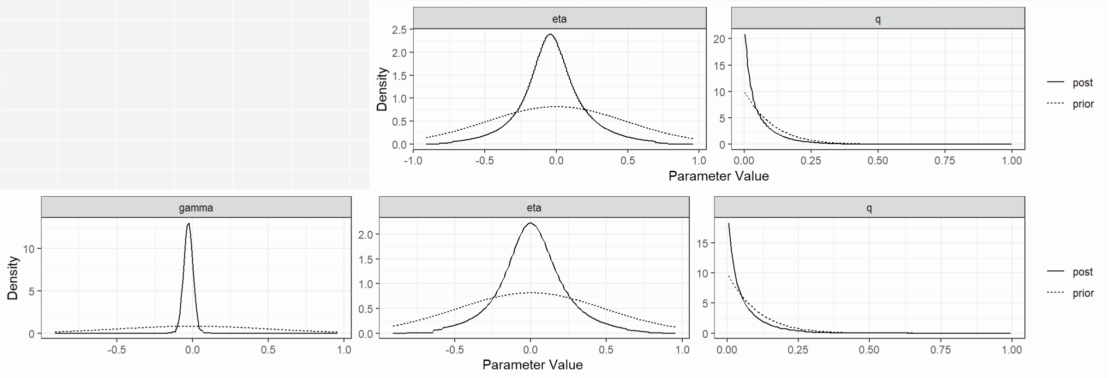

Figure S14. CAUSE-based MR analysis between genetically predicted EOC and COVID-19 hospitalization, related to Figure 2. Model 1 and model 2 refer to the models being compared (null, sharing, or causal). Model fit is measured by Δ Expected Log Pointwise Posterior Density (ELPD); Negative values of z indicate that model 2 is a better fit. Delta_elpd: Estimated difference in ELPD. se_delta_elpd: Estimated standard error of delta_elpd, and z: delta_elpd/se_delta_elpd.

	model1	model2	delta_elpd	se_delta_elpd	z	p
1	null	sharing	0.280	0.14	2.000	0.98
2	null	causal	0.093	1.10	0.087	0.53
3	sharing	causal	-0.190	0.94	-0.200	0.42

model	gamma	eta	q
Sharing	NA	-0.11 (-0.83, 0.69)	0.04 (0, 0.26)
Causal	-0.06 (-0.14, 0.02)	-0.02 (-0.76, 0.73)	0.04 (0, 0.25)

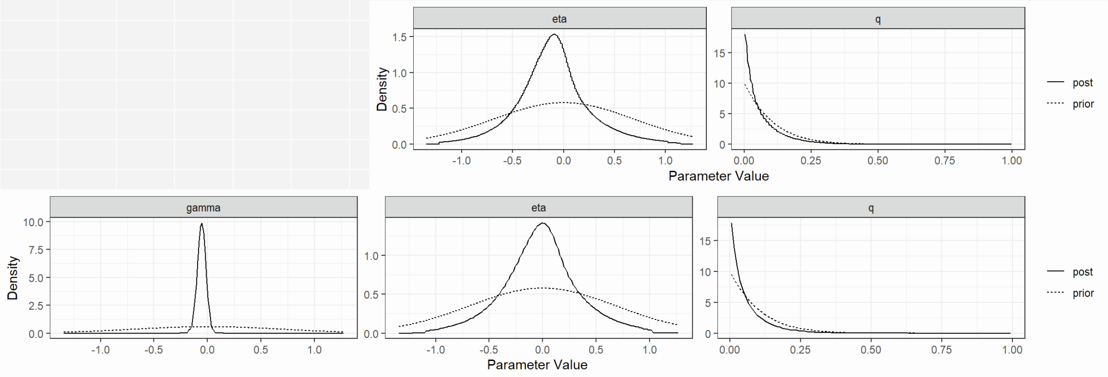

Figure S15. CAUSE-based MR analysis between genetically predicted EOC and COVID-19 critical illness, related to Figure 2. Model 1 and model 2 refer to the models being compared (null, sharing, or causal). Model fit is measured by Δ Expected Log Pointwise Posterior Density (ELPD); Negative values of z indicate that model 2 is a better fit. Delta_elpd: Estimated difference in ELPD. se_delta_elpd: Estimated standard error of delta_elpd, and z: delta_elpd/se_delta_elpd.

	model1	model2	delta_elpd	se_delta_elpd	z	p
1	null	sharing	0.48	0.18	2.6	1
2	null	causal	1.50	0.27	5.5	1
3	sharing	causal	1.00	0.10	9.8	1

model	gamma	eta	q
Sharing	NA	-0.01 (-0.38, 0.32)	0.03 (0, 0.21)
Causal	0 (-0.02, 0.02)	-0.01 (-0.34, 0.3)	0.04 (0, 0.25)

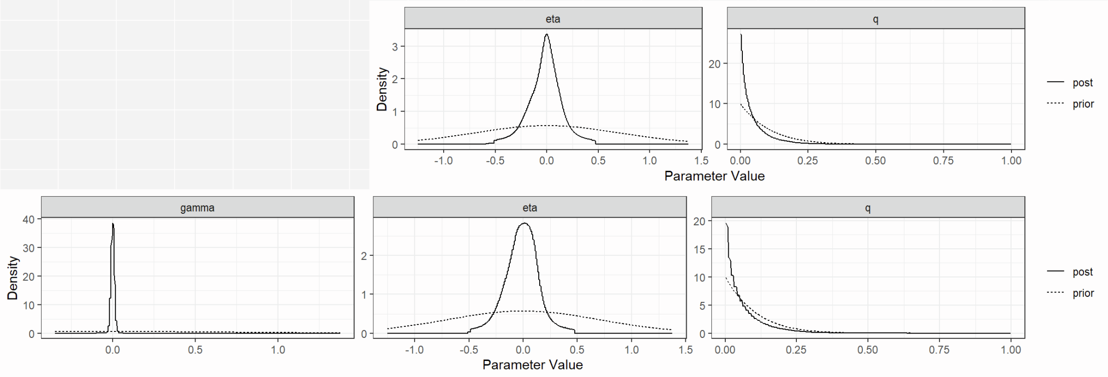

Figure S16. CAUSE-based MR analysis between genetically predicted EC and SRAS-CoV-2 infection, related to Figure 2. Model 1 and model 2 refer to the models being compared (null, sharing, or causal). Model fit is measured by Δ Expected Log Pointwise Posterior Density (ELPD); Negative values of z indicate that model 2 is a better fit. Delta_elpd: Estimated difference in ELPD. se_delta_elpd: Estimated standard error of delta_elpd, and z: delta_elpd/se_delta_elpd.

	model1	model2	delta_elpd	se_delta_elpd	z	p
1	null	sharing	0.38	0.26	1.50	0.93
2	null	causal	0.83	1.10	0.77	0.78
3	sharing	causal	0.45	0.86	0.52	0.70

model	gamma	eta	q
Sharing	NA	0.1 (-0.56, 1.16)	0.03 (0, 0.24)
Causal	0.02 (-0.02, 0.07)	0.01 (-0.6, 0.77)	0.04 (0, 0.25)

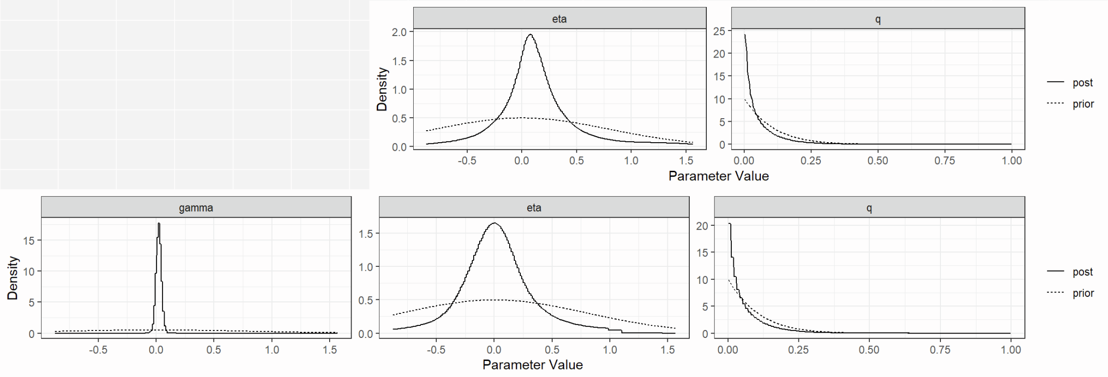

Figure S17. CAUSE-based MR analysis between genetically predicted EC and COVID-19 hospitalization, related to Figure 2. Model 1 and model 2 refer to the models being compared (null, sharing, or causal). Model fit is measured by Δ Expected Log Pointwise Posterior Density (ELPD); Negative values of z indicate that model 2 is a better fit. Delta_elpd: Estimated difference in ELPD. se_delta_elpd: Estimated standard error of delta_elpd, and z: delta_elpd/se_delta_elpd.

	model1	model2	delta_elpd	se_delta_elpd	z	p
1	null	sharing	0.42	0.21	2.0	0.98
2	null	causal	1.30	0.34	3.8	1.00
3	sharing	causal	0.86	0.18	4.9	1.00

model	gamma	eta	q
Sharing	NA	-0.04 (-0.95, 0.97)	0.04 (0, 0.22)
Causal	0 (-0.07, 0.07)	-0.05 (-0.9, 0.77)	0.05 (0, 0.29)

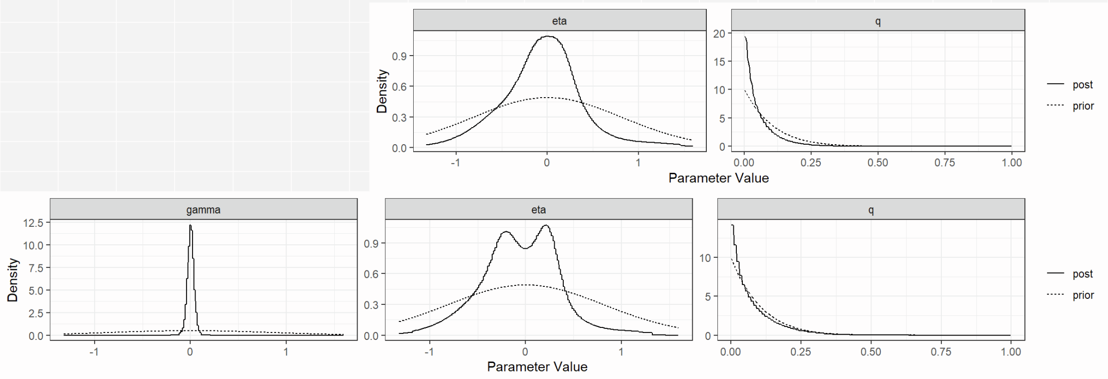

Figure S18. CAUSE-based MR analysis between genetically predicted EC and COVID-19 critical illness, related to Figure 2. Model 1 and model 2 refer to the models being compared (null, sharing, or causal). Model fit is measured by Δ Expected Log Pointwise Posterior Density (ELPD); Negative values of z indicate that model 2 is a better fit. Delta_elpd: Estimated difference in ELPD. se_delta_elpd: Estimated standard error of delta_elpd, and z: delta_elpd/se_delta_elpd.
